# Correction: Perceptions of risk for COVID-19 among individuals with chronic diseases and stakeholders in Central Appalachia

**DOI:** 10.1057/s41599-021-00974-9

**Published:** 2021-11-16

**Authors:** Manik Ahuja, Hadii M. Mamudu, Florence M. Weierbach, Karilynn Dowling-McClay, David W. Stewart, Manul Awasthi, Timir K. Paul

**Affiliations:** 1 College of Public Health, East Tennessee State University, Johnson City, TN 37614 USA; 2 College of Nursing, East Tennessee State University, Johnson City, TN 37614 USA; 3 College of Pharmacy, East Tennessee State University, Johnson City, TN 37614 USA; 4 Quillen College of Medicine, East Tennessee State University, Johnson City, TN 37614 USA

**Keywords:** Medical humanities, Health humanities

Correction to: *Humanities and Social Sciences Communications* 10.1057/s41599-021-00906-7, published online 7 October 2021.

The original paper lacked a clear statement on ethical approval and informed consent. The following statements have now been added to the paper to provide additional clarity for the reader:

Ethical approval

The research is part of an East Tennessee State University Institutional Review Board PCORI approved protocol that embodies the protection of human subjects.

Informed consent

The research is part of an East Tennessee State University Institutional Review Board PCORI approved protocol that embodies informed consent.

